# Collaborative Workshops for Community Meaning-Making and Data Analyses: How Focus Groups Strengthen Data by Enhancing Understanding and Promoting Use

**DOI:** 10.3390/ijerph16183352

**Published:** 2019-09-11

**Authors:** Barbara L. Allen, Johanna Lees, Alison K. Cohen, Maxime Jeanjean

**Affiliations:** 1 Department of Science, Technology and Society, Virginia Polytechnic Institute and State University, Blacksburg, VA 22043, USA; 2 Centre Norbert Elias (UMR 85 62), Ecole des hautes études en sciences sociales, 13236 Marseille, France; leesjohanna@gmail.com (J.L.); max.jeanjean@gmail.com (M.J.); 3 Department of Public and Nonprofit Administration, University of San Francisco, San Francisco, CA 94117, USA; akcohen@usfca.edu

**Keywords:** community-based participatory research, data interpretation, environmental health, knowledge justice, public health, participatory science

## Abstract

Community-based participatory research is a growing approach, but often includes higher levels of community engagement in the research design and data collection stages than in the data interpretation stage. Involving study participants in this stage could further knowledge justice, science that aligns with and supports social justice agendas. This article reports on two community-based participatory environmental health surveys conducted between 2015 and 2019 in an industrial region near Marseille, France, and focuses specifically on our approach of organizing focus groups to directly involve residents and community stakeholders in the analysis and interpretation process. We found that, in these focus groups, residents triangulated across many different sources of information—study findings, local knowledge, and different types of expert knowledge—to reach conclusions about the health of their community and make recommendations for what should be done to improve community health outcomes. We conclude that involving residents in the data analysis and interpretation stage can promote epistemic justice and lead to final reports that are more useful to community stakeholders and decision-makers.

## 1. Introduction

### 1.1. Participatory Data Analysis and Interpretation

Community-based participatory research (CBPR), in which people affected by the issues being studied participate in the research process, is on the rise [1] around the world, although most of this approach’s growth has been in the United States of America (USA). This article documents a case study of one way in which community-based participatory research can lead to more meaningful research by engaging community members deeply in data analyses and interpretation. This also has implications for the use of collaboratively created research evidence for broader public awareness and potential policy impacts in promoting environmental health [2].

One of the key principles of CBPR is attention to collaboration with communities throughout all phases of the research process from problem definition and data collection to data analysis and interpretation (and, hopefully, action) [1]. As has been pointed out by Binet et al. [3], there appears to be less engagement of participants in the data analysis and interpretation phases of a CBPR project. A 2003 survey of 25 CBPR projects revealed that while almost all projects involved participants in research design and participant recruitment, less than half (n = 9) included participants in interpretation of findings, and fewer yet (n = 4) included them in data analysis. The reasons for this range from longer timeframes, funding inflexibility, and institutional cultures, to lack of instructive examples on collaborative data analysis and interpretation [3]. Our research is an attempt to address the last issue by providing not only, a clear description of how we conducted collaborative analysis and interpretation, but also explain and illustrate why this last step is important.

We build on a relatively small history of doing such participatory data analysis within CBPR projects [4], examples include Binet et al. [3], Cashman et al. [5], Cohen et al. [6], Cohen et al. [7]. The most recent of those articles, Binet et al. [3], provides an in-depth description of how participatory data analysis unfolded in a CBPR study focused on neighborhood determinants of health in Boston. The oldest of those articles, Cashman et al. [5], provides brief case examples from multiple CBPR studies across the USA that involved community members in the data analysis and interpretation process. The other articles (Cohen et al. [6,7]) report on findings from a CBPR study that involved community members in the data analysis and interpretation process, and also reflect on how such work can support community organizing. To the best of our knowledge, these examples have occurred primarily in the USA, and this article is among the first examples of such work in France. For our collaborative workshops, we also build on the use of focus groups for understanding and meaning-making [8,9] as well as nurturing inductive reasoning [10]. Additionally, we add insights from Science and Technology Studies (STS). In particular, STS scholars posit that participation in science and collaborative analysis, especially by those for whom the science most matters, can strengthen scientific knowledge in several ways. Collaborative analyses can further epistemic justice [11] or knowledge justice [12], which is knowledge or science that aligns with social justice agendas in the wake of environmental health inequities. Through focus groups, residents are given space to make sense of the data from their lived experience in such a way that their views became more fully interwoven with their health data. Furthermore, they are able to suggest further statistical analyses, discuss causal hypotheses, and collectively suggest the next steps to be taken moving forward. Finally, the interweaving of qualitative and quantitative data further strengthens relevant science outcomes for the residents and better supports community use of the study and action.

### 1.2. Introduction to Case Study

In 2015, we began conducting a two-phase epidemiology-based community-based participatory health survey called Fos EPSEAL (Fos Étude Participative de Santé Environnement Ancrée Localement in French, direct translation in English: Fos Participatory Environmental Health Study Anchored Locally). The first phase was in two towns, Fos-sur-Mer and Port-Saint-Louis, in the industrial zone that is part of the larger port of Marseille, France. For years the residents and local doctors had asked for relevant health information from the French health service whose response was to conduct studies without resident input, most concluding that there were: few health problems in the region and/or that their study was inconclusive and more studies needed to be done [13]. Citizen frustration grew from the lack of relevant information. A petition requesting health data was circulated garnering more than 4000 signatures and several dozen residents participated in a sit-in at the French health service’s regional offices, only to be escorted off the property by riot police (for more details, see Allen et al. [14]). Our phase one study revealed numerous chronic illnesses that were much higher than the French population. In fact, elevated disease prevalences remained in direct age and gender standardized analyses we did [15]. We then held 28 focus groups with local residents to further analyze the data and to more comprehensively make relevant meaning from the sometimes-surprising statistical findings. 

This last step of collaborative analysis led to the production of a final report that effectively integrated quantitative data from the epidemiology survey with qualitative health data from the focus groups. By transforming data into lived experience in the qualitatively integrated report, it enabled ownership of the study by the local population. The result was substantial press coverage in the year following the study release, with over 150 news articles, radio shows, and TV segments. Both citizens and local public officials were featured in the various media clearly advocating for policies and positive change. 

For the second phase of the Fos EPSEAL project in 2018–2019, we conducted a similar health study in Saint-Martin-de-Crau, a heavy logistics/trucking and agricultural town on the periphery of the industrial zone. This town had been chosen for a comparative exposure study [16] by a local Fos-sur-Mer citizen science group, the Institut Éco-citoyen (IEC). Our phase two survey was, in part, in response to the residents of the industrial zone wanting some comparative health data from a town just outside of the zone. In conversation with the IEC and our key informants, we decided that align our study with their choice of comparison. After conducting that survey and doing preliminary data analyses, we held 19 focus groups, both in the two industrial towns and in the peripheral town. This provides a space for collaborative local meaning-making of the preliminary health outcomes and further analyses of the data. We argue that this final phase, collaborative analysis, lead to a greater community capacity via a stronger, more authoritative voice about population health that included consensus ideas about next steps.

## 2. Methods 

### 2.1. Study Methodology

We conducted participatory health studies using a CBPR approach and epidemiologic methods in three towns. Specifically, we conducted two cross-sectional health surveys of geographically-determined systematic random samples [15]. Our first sample (data collected in 2015) was drawn from two towns (Fos-sur-Mer and Port-Saint-Louis-du-Rhône) located in the heart of the industrial zone of the Étang de Berre region. Our second sample (data collected in 2018) was in Saint-Martin-de-Crau, a heavy logistics (trucking) and agricultural town, about 15 miles away and on the periphery of the industrial zone. In Fos-sur-Mer and Port-Saint-Louis, we surveyed 816 respondents, and in Saint-Martin-de-Crau, there were 439 respondents, representing approximately 3% of the population in each town. Quantitative data were analyzed to report descriptive and analytic statistics. More information is available elsewhere (see Allen et al. [17], Cohen et al. [15], Lees et al. [18,19]) about this process and these findings. The study, including the semi-structured focus group protocol, was approved by the Virginia Tech Institutional Review Board. Briefly, health issues including skin problems, asthma, eye irritation, cancer, and diabetes were elevated in comparison to the French population [15]. 

### 2.2. Intermediate Process: Public Meetings for Initial Data Dissemination

We presented the preliminary data from our first study [15] in open, well-advertised public meetings in Fos-sur-Mer and Port-Saint-Louis in June 2016. In the 2019 health outcomes study of the peripheral industrial and agricultural town of Saint-Martin-de-Crau, there were also surprising findings. We held an initial public meeting there as well, following our approach in the earlier town studies.

**Figure 1:**
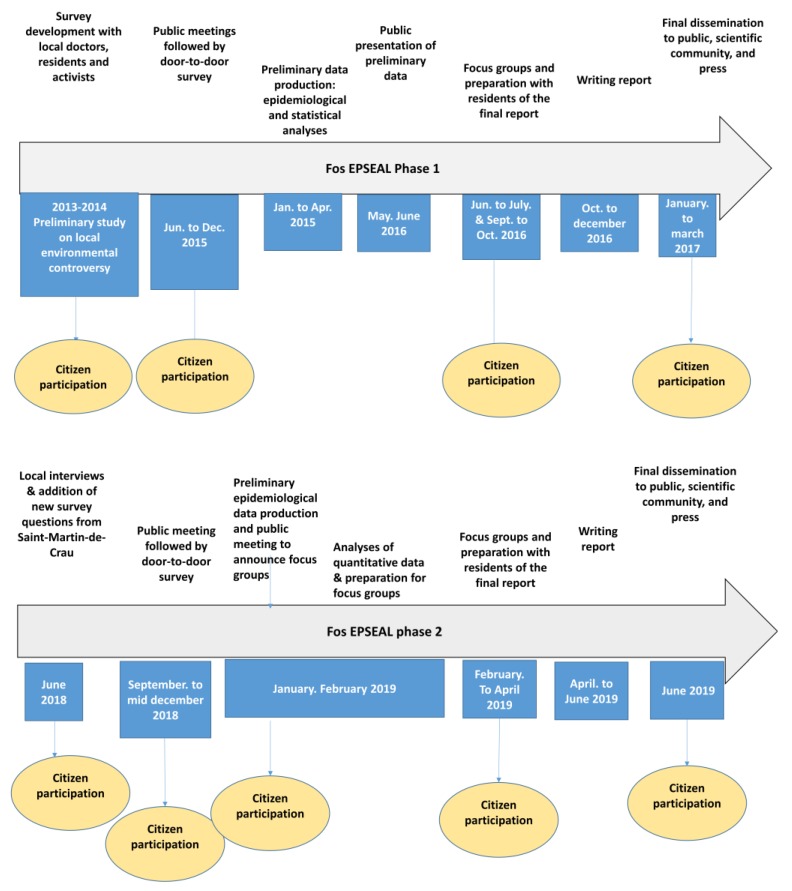
Study timeline and participatory elements of the process.

At all of our public meetings to disseminate our preliminary findings (June 2016 for phase 1, January 2019 for phase 2), we ended our presentations with a request that citizens volunteer to participate in a series of focus groups to further analyze the data and suggest next steps, given their new health status information (see Figure 1).

### 2.3. Collaborative Analyses of Data in Focus Groups

Between June and December 2016, we conducted 28 focus groups in Fos-sur-Mer and Port-Saint-Louis, with two to four occurring each week (focus groups were led by Lees and colleagues). The number of attendees varied from 1–10. All survey participants and other interested residents who voluntarily gave us their email address or cell phone number (collected and stored completely independently from any health data) were invited to the collaborative workshops. Additionally, we announced the workshops on our website [20] and to all key informants and civic environmental associations who worked with us leading up to the survey. We had a mailing list of more than 800 contacts. Each week we sent by email or text message the day, time, and address of the focus groups. Then, in phase 2 of the project, after the Saint-Martin-de-Crau initial health data were produced, we also held 19 focus groups between February and April 2019, in the towns (focus groups were led by Lees and Jeanjean and colleagues). Since residents in Fos-sur-Mer and Port-Saint-Louis were interested in comparing their health to a town peripheral to the industrial zone, we also held focus groups there. Additionally, we held two other focus groups: one was an initial group held with our key informants from both phase 1 towns and the other was with labor union health activists interested in reporting the outcomes to their members. The number of attendees in the phase 2 focus groups ranged from 1 to 13 (see Table 1 and Table 2). In these focus groups, a semi-structured protocol was used in which researchers participated as little in the discussion as possible, except to facilitate conversation and provide answers to any factual questions, to avoid any potential influence of the researchers over focus group participants. No incentives were offered to participate in the focus groups (besides free snacks), and it is unlikely that there was any social desirability bias. 

The collaborative workshops took place in two phases. In the first phase, we were able to submit the initial results of the study to participants who, based on these results, could ask the epidemiologists (Cohen and Jeanjean) for further statistical crosschecks. This often involved a narrative summary of the first epidemiological results as presented during the first phase of the collaborative workshops and then discussed with the participants in the working groups. For example, residents had been particularly interested in cancer going into our study. At the collaborative workshops, we reported that there was an elevated prevalence of cancer (crude prevalence and direct standardized) in our study sample in comparison to the prevalence of cancer in France and that the disparity in the prevalence of cancer between our study sample and France was particularly pronounced for women. These preliminary findings were presented by one of our focus group leaders in a presentation, during which participants were able to question and discuss the data. In these focus groups, residents often offered interpretations for how the findings aligned (or did not align) with their lived experiences, and sometimes also identified new hypotheses of interest. In this instance, they were interested in gender differences and in understanding the prevalence of different types of cancer. This often required negotiating with what was epidemiologically possible to analyze, due to limitations of statistical power and maintaining anonymized data.

At the end of the first two months of focus groups (June–July 2016) in the two industrial towns, the team had a list of kinds of new statistical analyses, as well as a request to have some specific themed collaborative groups. This second set of focus groups took place after France’s customary summer vacation period, in September–October 2016. The idea of themed focus groups emerged from the earlier group discussions, and we invited medical specialists and other experts to participate where appropriate. The residents were then able to reflect on their lived illnesses, observations regarding pollution, and hypothesize about causation in conversations with professionals.

The themes discussed during the fall 2016 focus groups included: asthma and respiratory disease, cancer, diabetes, reproductive problems, air pollution, weather conditions, and occupational exposures. Typically, we invited one expert to each meeting depending on the theme and they ranged from medical experts such as oncologists, reproductive specialists, and pulmonologists to air pollution scientists and trade unionists involved in occupational disease assessment.

In total, for the first Fos EPSEAL study of health outcomes in the two industrial towns, almost fifty people worked collectively to analyze epidemiological data. The open voluntary focus groups ranged from 1 to 10 participants that can be categorized into three groups: survey participants, specialists, and activists (see Table 3). Most of the focus group attendees (n = 30) had participated in the door-to-door survey and separately gave their contact information to be contacted to be recruited to participate in the focus groups. Some came once, and others, two or three times, with a few following the process to the end. Another group (n = 8) were medical specialists, general physicians, or air pollution measurement engineers, invited at the request of participants in collaborative workshops. These experts came one or two times to the focus groups. The last category (n = 10) of participants were from civic associations, local unions, or were environmental activists. 

Two team members typically attended the focus groups so that one could take notes and the other could lead the group. These focus group sessions typically lasted 1–2 h. The meetings began by introducing ourselves and having everyone in the room do the same. We would initiate a discussion with some basic rules such as the confidentiality of the participants’ names and/or any intimate information that may be shared. Additionally, we asked that the participants be respectful of each other’s views (i.e., by not interrupting or speaking harshly) and allowing time for everyone to speak. We would then give a presentation of the data so that there was baseline knowledge of the data. For the initial general focus group meetings, this was an overview of the health outcomes, for thematic meetings, this was a presentation of more in-depth health data from our study on that topic (i.e., asthma, cancer, diabetes, etc.). Finally, the team leader would initiate the discussion with questions such as: Do these data make sense? What feelings do the data evoke? Are you surprised by anything and why? The objective of the general questions was to initiate a conversation with the participants. 

During the focus groups, participants often discussed their ideas about the causes of their health problems and asked the team to check the scientific literature for any studies that might prove or disprove their hypotheses. As some of the themed groups were iterative, between each meeting, the research team read the scientific literature on the topics discussed and the hypotheses proposed and presented a synthesis of the research for participants in the next focus group. Reviewing the scientific literature allowed residents to validate or invalidate their previous hypotheses. This iterative process-built residents’ confidence in their expertise. The invited specialists were also further informed on the nature of illnesses in the community. The review of the scientific literature allowed focus group participants to build a solid analytical frame of the health and pollution context of the industrial zone they lived in.

Phase two of our Fos EPSEAL study occurred two years later in 2018–2019 in response to requests from the residents for comparative health data. We also had encouragement from an environmental science civic association in the region, the IEC, to conduct our study in the same town they had previously done an exposure study, a logistics and agricultural town on the periphery of the industrial zone, Saint-Martin-de-Crau (about 15 miles away from the original two study populations). Once we presented the new preliminary health survey data in a public meeting in Saint-Martin-de-Crau in January 2019, we held a total of 19 focus groups there and in the original phase one towns of Fos-sur-Mer and Port-Saint-Louis. With the comparative health data, the local population further made sense of the health outcomes of their broader industrial-agricultural region. There were 46 participants in the focus groups and some people came numerous times. The types of participants were consistent with the first phase study in terms of including citizens that came multiple times, invited doctors and other experts, as well as a civic association member and other local activists (see Table 4). 

We also engaged the focus groups for their advice on how we presented the data. This enabled us to have the clearest representation of the community’s health statistics when we produced the final report for the public. Finally, the last part of each group we dedicated to asking them to discuss what the next steps or action recommendations for the community, the state, and the local invited experts might be given the science they are helping make. These next steps were included in the final public reports that were put online after the public meetings.

## 3. Results

Overall, we found that people sought to make sense of the statistical analyses by comparing them to their own lived experiences and identifying areas of congruence and non-congruence. Residents were also interested in how the study findings intersected with other published peer-reviewed literature on the topic and the insights of local experts. Ultimately, residents, with the assistance of team members, triangulated across all of these different sources of information—study findings, local knowledge, and different types of expert knowledge—to reach conclusions about the health of their community and make recommendations for what should be done to improve community health outcomes. 

### 3.1. Statistics Do not Speak, People Do

In June 2016, at the end of an open public meeting where we presented the preliminary health outcomes in the industrial town of Port-Saint-Louis, one of the residents raised his hand. Expressing concern that the health study was not concluding with numerical tables, he said, “This data is not us—our lives are not charts and figures.“ He and others in the audience wanted to understand what this data meant for their lives and how this was inflected in the lived history of their communities. We explained that the health study was not over, that preliminary dissemination of the data was only a first step in preparing the final report, and that we would be holding a series of focus groups (called “ateliers” in French, or workshops) to enable local people to collaborate in analyzing the epidemiological data. These groups would be opportunities for residents to come together in small groups and discuss the data together in an effort to make sense of the numbers and their meaning in their daily lives.

One example was the surprising, potentially perceived to be contradictory, finding in our comprehensive health question in the two industrial towns. While we found that at least 63% of our respondents reported at least one chronic disease (as compared to 40% of the French population), 72% of the respondents self-reported their health as either excellent or good. Making sense of this in group discussions, the participants arrived at the idea that they normalized their chronic illness by managing their condition with medical treatment or otherwise learning to live with their conditions, which likely affected how they self-reported their general health status. “I am artificially healthy,” declared one resident, as she described taking numerous medications each day to feel well. Other factors also contributed to the residents’ optimistic health perception. For example, the severity of the disease considered and its impact on their daily life was part of the discussion. Living with a life-threatening cancer was perceived differently by the participants than managing chronic asthma. During the focus groups, the participants questioned whether their ongoing concern for health, given their degraded environment, has led to extensive sharing of health conditions with others in the town, and thus reinforced the fact that negative health outcomes are commonplace, even normal. On an optimistic note, some participants suggested that the positive self-reported health assessment was a reflection of the resilience of the residents and the perception that they were living on the sea, close to nature, with strong local community ties, and this somewhat negated their thoughts about industrial pollution.

To further illuminate the process and hermeneutical work of the focus groups, we have chosen two themes that emerged regularly from the discussions: respiratory illness and cancer. While other health concerns were discussed (i.e., fertility problems, chronic eye and skin ailments, and type 1 diabetes), it was these two health outcomes that seemed to elicit the most conversation for purposes of explaining the effectiveness of collaborative data analyses. 

### 3.2. Making Sense of Asthma and Respiratory Illness

Respiratory issues were a frequent topic of discussion in the focus groups. The group discussions followed two sense-making trajectories of causation/explanation and lived experience. First, participants hypothesized as to why adult asthma and other respiratory illness prevalence were elevated. (nearly sixteen percent of adult respondents had asthma, compared to 10.2% of the French population. Over 40% of the adult population and 23% of the children suffered from at least one form of chronic non-asthma respiratory illness including bronchitis, emphysema, and respiratory allergies not related to hay fever, as compared to 7% of the French population.) In our study, we found that 48% of the respondents with asthma said their illness began in adulthood, which is less common since asthma begins most often in childhood and, over time, these and related respiratory illnesses are less pronounced in adults [21]. Our study was cross-sectional in nature and so did not attempt to make epidemiologic claims about causality. However, while it was not possible to make a deductive case, residents were interested in making an inductive case drawing from different types of evidence. For example, our findings were unlikely due to smoking, since the prevalence of smoking in our study (30%) was slightly less than French prevalence (34%). Instead, numerous discussions focused on their hypotheses about environmental causation. Some participants postulated that the high level of air pollution was most likely a prime culprit, which was also supported by the research literature. (Often, after each focus group, team members would also comb the research literature to help answer any more specific questions or hypotheses residents had.) For example, we found that some scientists suggest that atmospheric pollutants have a role in triggering adult-onset asthma [21]. A retired general practitioner physician living in Port-Saint-Louis also hypothesized that the local climate and vegetation form a strong allergenic background noise, which amplifies the impacts of concentrated industrial air pollution in the towns. 

Other focus groups were interested in the spatial distribution of cumulative asthma in the towns. We had collected our data linked to a map, with collection districts roughly correlated with the neighborhoods in each town. In presenting a map of the relative prevalence of asthma in the towns to the Fos-sur-Mer groups, two neighborhoods in their town stood out. While the prevalence of asthma in adults in Fos was higher everywhere than the French prevalence, two non-contiguous neighborhoods exceeded 20% prevalence. In discussions, the participants suggested that main roads with significant truck traffic bound the neighborhood on the far north and west of the town. Additionally, the area was exposed to industrial pollution regardless of which way the wind blew. This hypothesis was confirmed by an engineer from Air PACA, the government-funded air monitoring association invited to the focus group. The second Fos neighborhood on the eastern edge of the city bordering the Étang de Berre, a highly industrialized lake of brackish water, also had elevated asthma. The residents had varying hypotheses for this. One idea was the closeness of the cement plant and a major road or potentially the pollen profile of the area.

A pneumologist from Arles, who had once practiced in Port-Saint-Louis, attended, a group focused on asthma and respiratory illnesses. She was verbally working out her understanding of the asthma phenomena as she participated in the discussion:

It is not surprising to find more respiratory diseases in a region where there are factories because there are emissions- that makes sense. But there are things I cannot put into words and I thought of coming to your working groups. What is interesting for me in your study are the nose-throat symptoms, which are not usually listed and I have the impression that in twenty years we will say that they are precursors of certain diseases.

The second sense-making trajectory was a group discussion of the lived experience disease. This included sharing experiences of living with illnesses to inform a more holistic understanding how it shaped the contours of daily life.

Participants explained how their bodies and lives are disrupted during ‘bad air days’:

I have been on cortisone for the past fifteen days [when an air alert was in effect] and I could not go out of my house. If the focus group had taken place yesterday, I could not have come as there was no wind and it was unbreathable. I get coughing fits in my sleep and am awakened…sometimes I am afraid as I cannot seem to take a breath.

*(Asthma group participant)*

In July 2015 during an air alert period, I had trouble breathing. It was a crisis for me and the fireman had to come to my house. It was during the holidays.

*(Fos-sur-Mer participant)*

I have a tingling in my throat and choking sensations at night with difficulties swallowing. It happens at least once a month when there is no wind to blow the pollution away.

*(Fos-sur-Mer participant)*

The impossible cohabitation between outdoor exercise and industrial practices was a point of frustration and adaptation. A cyclist mentioned that he puts his bike in his car and begins his touring twenty to thirty kilometers north of the city. A jogger noted that, on cloudy days, it is more difficult to breathe at the end of his usual loop than in clear weather. Explains another Port-Saint-Louis participant: “We are advised not to do too much sport when it is polluted, but it is hard to live that way”.

There are also other ways that the townspeople dealt with the heavy load of pollutants on their bodies. Numerous participants mentioned going to cleaner environments for vacations as much as possible—and how well they could breathe and how much better they felt when they came back. One participant said (echoing others), “We go twice a year for two weeks in the Pyrenees, and twice a year for two weeks in the southwest too—we go there to breathe”. Another respondent explained that, “we have to go to the mountains and when we’re there, it’s better, and we breathe-- and as soon as we come back here, we feel the difference’”. Through group discussions, we discovered that vacationing in the mountains or countryside far from the industrial zone was a common coping strategy for residents living with respiratory illness.

### 3.3. Making Sense of Cancer

Cancer was another common topic among participants trying to make sense of the health data. While the prevalence of cancer in the towns was 10.5% compared to 6% for the French population, it was much higher for women (14.5%) as compared to men (8.3%) in our study. Participants discussed the endocrine-disrupting nature of some of the industrial pollutants as a possible reason for elevated cancers in hormone-dependent areas of the body, noting that thyroid cancer was also elevated. They also hypothesized that women were more likely to seek medical care for symptoms, thus may receive earlier, and potentially more, diagnoses for health problems.

Noting the absence of certain cancers with poor prognoses (i.e., lung and pancreas), focus group participants wondered whether men might be more impacted by these diseases. Such discussions provided opportunities to discuss limitations of an epidemiologic approach (for example, we explained that their absence does not mean they do not exist, but that either our numbers were too low for inclusion (i.e., less than ten cases for privacy and statistical reasons) or that people with these illnesses were too sick to answer our survey) and allowed our focus group leaders (who also did door-to-door surveying in the first study) to draw upon their own lived experiences. According to the surveyors, almost everyday day someone would respond, “I am too ill to speak with you”, or, “It’s too difficult to talk as my husband is in the middle of dealing with cancer”, or, “Thank you mademoiselle, but I am seriously ill and do not want to talk about it”.

Another statistic that stood out in the study was that 15% of residents reported having or having had more than one type of cancer (two to four cancers per person). While cumulative numbers of cancers in the same person can happen, the doctors, including an oncologist, in our groups felt that the frequency of this phenomenon in the study, as well as the nature of the cancer sites, seemed atypical. A retired local physician who attended some of the focus groups explained his professional view:

You have different stages of illness in this area. First, there are the immunological diseases (lupus, vascularites/phlebitis, skin disease, respiratory illness, chronic bronchitis) followed by cancers such as immunological cancers, and then sarcomas of the lung, lymph, and different kinds of pharynx/throat cancers. Finally, in some cases, the same person has a multiple cancers and/or cardiac disease or attacks. My wife (also a local physician) and I consider that there are three stages in time for a pathology here, on a period of twenty years: (1) Small incidents stage; (2) Strange disease stage where it looks like the disease you’ve studied in text books, but with always a little difference, and (3) Catastrophic stage with cancers, strokes.

Another focus group participant in Fos-sur-Mer remarked about what she had observed in her community over the years:

It’s true that in the neighborhood there was a lot of illness. One of our neighbors had two sons both die of cancer at about thirty years old. One had a brain tumor, the other I don’t know. On our street there were other brain tumors. I do not know if it’s a coincidence, but it’s true in our neighborhood that a lot of cancer exists and it really worries us.

A 23-year-old student who had always lived in Fos-sur-Mer attended a group and shared his story:

I have a family of cancer patients. I have two uncles who died of pancreatic cancer, one of them was 30 years old. My grandfather, he died of cancer too, he was working in Lafarge, and my cousin he died of skin cancer he was 30 years old too. I have two cousins who have breast cancer and one had to remove her breast.

In our second study, in the logistics and agricultural town of Saint-Martin-de-Crau, our findings were surprising. In general, while the population was somewhat healthier than the residents of the industrial towns, their health outcomes were still worse than the French population. Additionally, their cancer incidence (12.4%) was comparable to the industrial towns (10.8%) (this difference was not statistically significant). We explained the concept of confidence intervals and the limits of the state comparative data that we were using in the focus groups, so they were aware that statistics were not precise, but had a degree of play in terms of actual prevalence. While the participants said they understood this they discussed why their cancer rates were even the same as Fos-sur-Mer, given their assumption that it would be much lower as they were on the outskirts of the industrial zone. 

In making sense of the cancer data for the logistics and agricultural town, the focus groups generated several hypotheses. Besides having similar, though less intense industrial air pollution than the two industrial towns, Saint-Martin-de-Crau residents likely have more exposure to pesticides, both occupationally, and due to crop spraying, including fruit tree spraying from helicopters. One of the top 4 cancers was lymphomas and there was evidence in the literature of the relationship between pesticides and this cancer [22]. There was also the exposure to DDT from mosquito spraying, an exposure shared with the other two towns. The residents of all three cities mentioned skin cancer due to both sun exposure and the exposure of the skin to excessive chemicals in the air. 

A notable difference in the cancer outcomes of the two studies was the number of people diagnosed with more than one cancer. In the two industrial towns, 29.3% (Port-Saint-Louis) and 16.7% (Fos-sur-Mer) of people who had cancer had 2–4 types of cancers in their bodies, compared to only 6.4% of those with cancer (and never more than two cancers) in Saint-Martin-de-Crau. Interestingly, the idea to look at cumulative cancer came from a focus group discussion. The participants wondered why there was such a high incidence of cancer accumulation in Port-Saint-Louis compared to the other two towns in the studies. They recalled that industrial activity began in the early 20th century in Port-Saint-Louis compared to the 1960s in Fos. They also mentioned that other citizen science efforts, such as that by the IEC, has shown greater soil contamination in or near that town due to older industries that have since closed. Several oncologists attended various group meetings and explained the complexity of associating multiple cancer diagnoses in individuals. Commenting on multiple cancers being discussed, a local oncologist explains to the participants:

The association of cancers such as breast and ovarian is not surprising. There are cancers that are induced, for example, a patient who has had a melanoma and had chemo-this can lead to another cancer such as a lymphoma.

The oncologist continues to speak of other chemo and radiation-induced cancers and is questioned by a participant about the seemingly large number of cancers that are environmentally linked. She continues:

Yes, that’s a lot. What seems to be happening is that there may be a few more ‘cancer’ associations here than elsewhere but it’s not necessarily environmental as people may have had several cancers related to the environment and after that the treatment of the cancers may have led to more.

What is clear is that there is fear and stress among the population around cancer. In a number of focus groups, stress was considered a major impediment to residents’ well-being. The air quality caused stress and the fear, particularly of cancer, loomed large. A resident of Fos explains:

As soon as we are sick, we are afraid it will be cancer. I know many people who have cancer. I have a colleague that died at 45 and two other colleagues suffering from cancer including breast cancer. More than we say ‘I have the flu,’ we say ‘I have cancer.’ It’s scary.

One inhabitant in a focus group remarked that “here when we say someone has died a natural death, we mean they have died from cancer.” Another backed up his comment by remarking that, “here it is a living cemetery.” 

### 3.4. Results of Collaborative Analysis: Participant Recommendations

Toward the end of each focus group, we encouraged the participants to reflect on the next steps they or others might take given their understanding of the health data.

During the first phase, the focus group participants had several main recommendations. The first was about disseminating the report and the health results to the press. We held a press conference and also answered all journalists’ questions if they contacted anyone on the team. In the meantime, some residents sent the report to the media as it was accessible online. More than 150 articles were published about the study in local and national newspapers, including a few television documentaries about the zone and numerous radio features about health problems in the region.

Residents also had recommendations related to environmental regulation. First, they recommended changing the legislation about air pollution and pollutant emissions to limit the pollution authorized in the industrial zone. They also wanted the government to assert more control over documenting air pollution rather than relying on the industry’s self-reported emissions estimates. Second, the focus group participants recommended local adaptations to the degraded environment. For instance, they wanted children to avoid outdoor playgrounds during pollution peaks and suggested building an indoor playground. They also wanted alternatives to blowers for street cleaning, since they added more particulate matter into the air. The residents also argued for policies that would encourage more rail traffic instead of truck traffic to help limit logistics-related pollution. 

In addition to recommendations regarding the environment, residents also had recommendations regarding access to healthcare. Residents argued that their health status was specific to being in the industrial zone, and so a local health care policy should meet those needs. They asked for a special health public policy to treat their health problems, which would entail having more primary care providers, more specialists, more hospitals, and more dispensaries in the industrial zone. They also wanted access to additional health screening. For instance, they argued, women should be screened for breast cancer earlier in the industrial zone than in France due to the elevated rate of cancer in the zone. They asked for access to better public transportation to local hospitals as they felt isolated. Also, they asked for the creation of a local cancer registry as well as a registry of chronic pathologies such as respiratory diseases, cardiovascular pathologies and diabetes. In general, the residents were interested in getting more information. They wanted the government to provide more frequent (specifically, daily) information about air pollution and wanted government information about how their environmental quality could affect their health.

They also had ideas for future research. For instance, they asked for more local data about occupational health within the industries and for more study about the cocktail effects of their combined environmental exposures. They also asked that a similar study be done in another town for the purpose of having comparable data with the same methodology. They also wanted the results of the Fos EPSEAL study analyzed with the results of an exposure study made by a local citizen science organization, the IEC. These last two requests were what inspired our own study’s phase two.

During the second phase of our study, we also asked residents participating in the focus groups for their recommendations, some were similar to the first phase. Residents continued to be interested in better air pollution regulations, better health policies, and replacing truck traffic with rail traffic. However, some recommendations were more detailed. For instance, residents suggested that industrial workers be monitored for health from the beginning of their career to the end. Considering air quality, they advocated for a policy measuring all pollutants, including ultrafine particles which are known to have negative health impacts. This would require local governments investing in tools to measure these particles. Residents appreciated having national and EU-level air pollution regulations but also argued that it was important to have regulations specific to the industrial zone. This was because they have a cocktail of emissions and current regulations regulate each pollutant separately and do not consider the cumulative effect of multiple pollutants simultaneously.

Some new ideas emerged in this second phase. One recommendation was to create an evacuation plan in the event of an industrial accident. They also wanted all of the local and regional governmental bodies to incorporate health risk prevention considerations and provide specific mechanisms to support families affected by cancer. As only 4 of the 46 phase two focus group participants had participated in the phase one groups, we would only lightly attribute the expanded recommendations to iterative attendance across both phases of the project. We, however, hypothesized another reason for their broader and more detailed thinking: the immense media coverage of the first survey, both in the region and nationally, which continued for well over a year. This kept health issues and proposed interventions in the forefront of public discussion in the industrial zone and participants several years later, in phase two, were the beneficiaries of this. These participants were similarly interested in disseminating the study findings broadly. They asked the team to send the Fos EPSEAL report to all local mayors, relevant government officials, and to the local industries. They also wanted the results disseminated to the students and teachers in local high schools. 

## 4. Discussion

### 4.1. Frame of Participatory Approach

First, by fully engaging residents in participatory science including the final analyses of the data engenders greater social relevance [12,23,24,25], and as some have argued, even better science:

*The more socially robust one’s knowledge claims, the more empirically reliable they will be. That is, the more scientific research projects engage with their social environments in egalitarian discussions, the higher the quality of the results of that research*.

*[26] (p. 97)*

Second, including local residents in the data analyses phase of a project can promote social justice by including the knowledge claims of the people who live and experience their compromised environment on a daily basis. This epistemic justice has two equally important parts of testimonial justice and hermeneutical justice [11]. According to Fricker:

*Testimonial injustice occurs when prejudice causes a hearer to give a deflated level of credibility to a speaker’s word, hermeneutical injustice occurs at a prior stage, when a gap in collective interpretive resources puts someone at an unfair advantage when it comes to making sense of their social experiences*.

*[11] (p. 1)*

In the focus groups, residents’ embodied knowledge and empirical observations are fully considered: they are deemed credible and their everyday observations and tacit knowledge [27] are taken seriously and form the basis of the group discussion. The collaborative analyses of data can alleviate the hermeneutical injustice perpetuated when residents are trying to fully grasp and give voice to complex environmental health issues. Poor and working-class communities, such as the ones in our study, can struggle to convey their experiences in a clear manner as if they were seeing the problem, “*through a glass darkly, with at best ill-fitting meanings to draw on in the effort to render them intelligible”* [11] (p. 148). The language of statistical data can exacerbate the injustice as it further alienates the residents in exactly the arena they would do well to understand and participate in for policy influence and action.

### 4.2. From Collaborative Analysis to Final Report

During the focus groups, participants were given the space to make sense of the epidemiological data from the perspective of their own lives and observations. Their observations and discussions, sometimes with medical professionals, were taken seriously and recorded by a team member. A few days after each focus group, a one-page synthesis of the group deliberations was emailed to all the participants who had attended at least once. All syntheses were immediately posted on the project’s public website [20] so that all residents, medical professionals, local officials, and anyone else who was interested had access. The syntheses were often referred to in successive groups to enable quick uptake of previous discussions. This led to further observations of living with pollution and illness and deeply contextualizing the data in terms of lived experience. Additionally, attendees hypothesized about the reasons for their health conditions and, if available, a team member would research the literature often providing further backing their suppositions.

Summaries of focus groups discussions, as well as some direct quotes, were woven into the narrative of the final public report alongside data charts. For example, the discussion of cancer in the focus groups was given eight pages in the public report which included easy-to-read graphics. The epidemiology statistics were presented in colored graphics, with confidence intervals, which were explained to the participants [18]. 

Team members had asked participants during the focus groups if the charts made sense and what recommendations they had for clearer graphic presentation. Opinions of the invited medical professionals and air pollution experts were also summarized in the report alongside short definitions of the various illnesses discussed. An annex at the end of the report summarized the literature we found supporting the residents’ and invited experts’ hypotheses. Finally, we also met with key informants during the writing of the first report to confirm that the language and flow of the report represented the voice of the residents in the groups.

In the final report, the quantitative and qualitative data were integrated narratively and graphically within each section. This hybridization of data in the public report had numerous positive outcomes. First, the participant residents were taken seriously, and their voices were included in the final report, remedying, in part, the testimonial injustice often observed in marginalized communities fighting environmental harms. Second, hermeneutical justice was demonstrated as the meaning-making focus group conversations enabled residents to make sense of data, giving them more confidence, clarity of speech, and full ownership of the study. This was evidenced by the many local people who talked to the media about the study outcomes:

*The impact of the Fos EPSEAL study has shown the media spotlight on Fos-sur-Mer, says Daniel Moutet, the president of the association ‘for the defense and protection of the Gulf of Fos’*.

*[28]*

*In the words of Mayor Raimondi ‘of Fos’, the media coverage of the Fos EPSEAL study and the numerous reports on the issue of pollution in Fos-sur-Mer broadcast during 2017, have awakened consciousness. . . The Fosseans (local residents) are finally taken seriously*.

*[28]*

*From now on, the residents of the industrialized zones of the Étang de Berre region will be able to brandish this study like a weapon in order to defend their interests and to weigh in on the local discussions*.

*[29]*

Third, situating the epidemiological data in the report alongside focus group hypothesizing, allowed lay-people’s reasoning about causation to be taken seriously. This was further substantiated in the final report by including citations of research that aligned with their theories. While our team did not make causal claims from the statistical health data, the residents and invited medical professionals were free to think outside of our data in the context of the community. Causality is hard to assess in environmental health research, in which environmental exposures are rarely randomized, so building this inductive case, while also limited, was nevertheless useful for residents. For the local population, the final report read in a normative way as a clear, coherent statement of health outcomes, the lived experience of illness in a compromised environment, and ideas as to why this may be happening to them. 

### 4.3. Positive Outcomes and Limitations 

The social integration of knowledge via collaborative analysis leads to a stronger and more relevant report compared to socially remote knowledge that is contained in official state studies or regulatory permitting documents [14]. It is counter to regulatory science that often does not speak to lay-people and the ways in which they live and navigate their neighborhoods and places of work. This enables them to be better advocates for policy, to speak to the press, and agency officials and build capacity for other actions.

After the release of the public report of Fos EPSEAL phase 1, the local agency for health, Agence Regional de Santé (ARS) asked its regulatory authority at the national level, Santé Publique France (SPF), to assess the report. Twelve specialists (sociologists, epidemiologists, historian) were mandated to assess the report and its methodology. The results of the assessment from SPF [30] was then disseminated in a meeting headed by the local subprefect, the state official in charge of public safety and risk. All local industries, local organizations, and state agencies were invited. Beyond criticism that some methodological information was missing from the public report (for which the residents were the primary audience), the report from SPF concluded that the Fos EPSEAL participatory study approach warranted merit and that further resident- inclusive studies about health in the industrial zone should be done.

Simultaneously, the French ministry of the environment also produced a report about health and environment in the industrial region [31]. For this report, the state agency interviewed our research team and summarized results related to epidemiological studies in the region. After those two reports from state agencies consecutive to the release of the Fos EPSEAL study, public meetings conducted by the subprefect continued to take place. In response, some government agencies launched studies, the most comprehensive one led by the local organization for the prevention of industrial pollution (SPPI, secretariat permanent pour la prevention de la pollution industrielle). This study sought to organize a process of citizen participation in the industrial zone, with the goal of communicating with the local population and responding to their concerns. It also sought to give the residents access to reliable information about their industrial environment and propel new actions related to both health access and improved risk knowledge of the local environmental context.

Another result that emerged from local and national government agency work was the decision to create a regional cancer registry. Local residents and organizations had been advocating for the creation of a cancer registry for more than 20 years. In 2019, during one of the subprefect’s meetings, the decision to create a local cancer registry, led by the local health agency (ARS), was made public. Nevertheless, some questions remained, including, if all types of cancers would be registered, if citizens and local organizations would be involved in the governance of the cancer registry, and why there would not also be a registry for other diseases that were elevated in the region (e.g., respiratory problems, type 1 diabetes).

### 4.4. Implications for Future Research 

While it is quite common for community-based participatory researchers to engage community members in the beginning stages of the research process (of identifying research questions and research approaches), engaging community members in the data analysis and interpretation phase remains all too rare. We encourage future researchers to learn from our example of conducting focus groups at scale, and to draw upon examples presented by others (e.g., Binet et al. [3], Cashman et al. [5], Cohen et al. [6,7]) to integrate community members into the sense-making stage of the research. Ideally, this will lead to developing a consensus on promising practices for participatory analysis, interpretation, and action in the future.

Focus groups are well-equipped to produce contextualized knowledge, but it requires a long-term process and commitment. Building this knowledge requires an iterative accumulation of information and triangulating across different sources of data, and this co-production of knowledge takes time. Indeed, before this process even begins, community members and researchers need to build trust toward building capacity to be able to analyze and interpret data together. Then, as part of iterative knowledge generation, this can include multiple cycles of hypotheses generation, analyses in response to those hypotheses, interpretation, and back again. Over time, with multiple cycles, these focus groups can lead to a refined understanding of health in environmentally distressed communities. 

## 5. Conclusions 

Promoting knowledge justice within CBPR can be resource-intensive, but we argue that it is worthwhile. To the best of our knowledge, this article reports on one of the largest-scale efforts to involve community members in the data analysis and interpretation phase of research. In our case study from the industrial zone of France, we found that community residents not only triangulated our study’s findings with their own local knowledge, but also eagerly sought to learn from the published, peer-reviewed academic literature, and local health and environment experts as they sought to make sense of the study findings and identify recommendations to improve the environmental health of the region. We encourage future researchers and practitioners to work together to facilitate collaborative sense-making through focus groups. 

## Figures and Tables

**Table 1:** Focus group attendance in phase 2 of Fos EPSEAL.

List of Focus Groups	Saint-Martin-de-Crau	Fos-sur-Mer	Port-Saint-Louis	Others
Focus group 1	13	4	4	3
Focus group 2	5	1	3	3
Focus group 3	8	5	3	
Focus group 4	2	4		
Focus group 5	6	1		
Focus group 6	5	6		
Focus group 7	8			
Focus group 8	6			

**Table 2:** Quantitative description of the iterative process of focus groups in phase 2 of Fos EPSEAL.

Focus Group Characteristics	Fos-sur- Mer	Port-Saint- Louis	Saint-Martin- de-Crau	Total	Other Groups
Number of participants	12	4	30	46	5
Number of participants who participated once	8	1	19	28
Number of participants who participated twice	2	1	5	8
Number of participants who participated three times	2	1	1	4
Number of participants who participated four times	0	0	0	0
Number of participants who participated five times	0	0	5	5
Number of invited participants	3	1	2	6
Number of weeks groups held	5	4	8	8	

Note: We did not store attendance data in a similar format for phase 1 of the study.

**Table 3:** Individual profile of some focus group participants in phase 1 of Fos EPSEAL.

Participants in the Focus Group	Description of Health Concerns in the Industrial Zone
**People recruited from door-to-door survey (age decade provided for anonymity purposes)**	
Female lifetime resident of Fos-sur-Mer (70s)	Did not express a particular health problem but had concerns about environment
Male resident of Fos-sur-Mer (20s)	Worker in the industry. Expressed concerns related to exposure at work leading to health problems
Male dock worker and lifetime resident of industrial zone (40s)	Worried about prevalence of disease in the industrial zone and especially about exposure at work. Declared during the focus group that he had respiratory problems in his childhood and back problems related to his job
Female high school teacher and lifetime resident of the industrial zone (40s)	Is concerned about health problems and pollution especially for the young generations
Female lifetime resident of the industrial zone (50s)	Had 3 cancers
Female resident of Fos-sur-Mer with her family living in the industrial zone for 3 generations (40s)	Father died from asbestos exposure and she is in the process of being diagnosed with auto-immune disease
Male, born in the industrial zone and lived there a total of 25 years (40s)	Previous dock worker with deafness problems and many concerns about his health
Female (30s)	Has cancer
Resident of Fos-sur-Mer (60s)	Has cancer and a 43-year-old son who works in the industrial zone
Nurse (50s)	Work at the local hospital and is concerned about the relationship between health and environment in the industrial zone
Male (40s)	In the process of diagnosed for pancreatic cancer
Female lifetime resident of the industrial zone (50s)	Has asthma and respiratory disease
Male born in Fos-sur-Mer and returns every summer for vacation (60s)	Concerned about pollution in the zone and knows many people in the industrial zone that are ill
**Experts and local officials**	
Deputy mayor of Port-Saint-Louis	In charge of the health issues for the city of Port-Saint-Louis
Local general practitioner doctors	Both arrived in Port-Saint-Louis 15 years ago from another part of France (Grenoble). Worried about the amount of illness in the town especially respiratory disease, diabetes type 1, and cancers.
Local pneumologist	Works in nearby in Arles as well as in Port-Saint-Louis
Local oncologist	Works at the local hospital and is interested in health questions especially cancers
Physician and reproductive specialist from Marseille	Studies infertility problems in the region of Fos-sur-Mer and in a nearby agricultural zone
Engineer from the local air quality monitoring organization (Air PACA)	Interested in air quality issues
Physician and epidemiologist from citizen science association in Fos-sur-Mer, Institut Éco-citoyen	Director of health and environment for the Institut Eco-citoyen and concerned about diabetes type 1 in the zone.
Director of the Institut Éco-citoyen	Conducting a comparative exposure study of residents in Fos-sur-Mer and another town (Saint-Martin-de-Crau) on the periphery of the industrial zone
**Activists from trade unions and local environmental organizations**	
Activist for the ADPLGF (Association de Défense et Protection du Littoral du Golfe de Fos, local environmental organization) and for the trade union CGT (Confédération Général des Travailleurs)	Concerned about infertility problems in the industrial zone
Activist for the CGT	Long-time activist for a local organization advocating for the recognition of occupational diseases in the industrial zone.
Activist for the CGT	Long-time activist for a local organization advocating for the recognition of occupational diseases in the industrial zone. Has lung cancer but never smoked
Activist for a health and environmental organization in the industrial zone	Concerned about health problems in the zone for many years
Activist for the ADPLGF	Concerned about health problems for many years
Activist for the ADPLGF	Concerned about health problems for many years
Activist for the ADPLGF	Concerned about health problems for many years
Activist for a health and environmental organization in the industrial zone	Concerned about health problems for many years
Activist for the ADPLGF	Concerned about health problems for many years
Activist for a health and environmental organization in the industrial zone	Concerned about health problems for many years and was very critical of the state-led health studies published in the industrial zone

**Table 4:** Summary profile of focus group participants in Phase 2 of Fos EPSEAL.

Focus Group Characteristics	Saint-Martin de Crau	Fos-sur-Mer	Port-Saint-Louis	Total
Total numbers of participants	29	12	4	45
Participated in the door-to-door survey	22	0	0	22
Participated in a focus group during Fos EPSEAL, phase 1	0	2	2	4
Works or has worked in industry in the industrial zone or has a relative working in industry in the industrial zone	15	6	1	22
Lived in the industrial zone	5	12	4	21
Is concerned about the industrial environment	10	7	4	21
Is concerned about the links between health and industrial environment	7	12	4	23
Is concerned about environment and agriculture	5	2	3	10
Is concerned about health problems related to pesticides	3	2	3	8
Is directed affected by illness or has a relative affected by illness	5	3	2	10
Participated in public meetings where preliminary data was disseminated	10	3	3	16
Trade union activist and/or local environmental organizations activists	0	4	3	7
Men	12	6	2	20
Women	18	5	2	25
